# Supplementary material for: Boosting Live Malaria Vaccine with Cytomegalovirus Vector Can Prolong Immunity through Innate and Adaptive Mechanisms
Source: bioRxiv. 2023 May 16:2023.05.02.539025. Originally published 2023 May 2. Preprint. [Version 2] doi: 10.1101/2023.05.02.539025 (PMC10187235; doi:10.1101/2023.05.02.539025)
Supplement: 1 [file NIHPP2023.05.02.539025V2-supplement-1.pdf]

S1 Fig. *Plasmodium chabaudi* MSP-1 B5 antigen-expressing MCMV vaccine vector.
(A) *P. chabaudi* merozoite surface protein MSP-1 (B5:1157–1171) B5 epitope is expressed as an MCMV immediate early gene. B5 epitope was introduced into an MCMV-K181 bacterial artificial chromosome (BAC) using recombineering. **(B)** The indicated organs were collected from MCMV infected mice at different times post- MCMV infection, homogenized, and MCMV titres were determined by plaque assay. The dashed line represents the limit of detection (L.O.D.) for each organ. Mean ± SEM are plotted with three mice for each time point. **(C)** Time course of qPCR assessment of MCMV infection in multiple organs. Mice were infected with MCMV-B5 or MCMV-BAC and organs were collected at indicated times for vector quantification with n=6 per time point. Copy number of CMV/β-actin (±SEM) ratio and L.O.D. of the assay are shown. L.O.D was calculated using data from uninfected animal. (**D**) Mice were infected with MCMV-BAC or MCMV-B5 and splenocytes were recovered 15 days later for MCMV-induced polyclonal T cell phenotype. Plots show gating and graphs show numbers of polyclonal $CD4^+$ T cells effector

bioRxiv preprint doi: https://doi.org/10.1101/2023.05.02.539025; this version posted May 16, 2023. The copyright holder for this preprint (which was not certified by peer review) is the author/funder, who has granted bioRxiv a license to display the preprint in perpetuity. It is made available under aCC-BY 4.0 International license.

($CD127^{-}$) and memory ($CD44^{hi}CD127^{hi}$) phenotype cells recovered after MCMV-B5 compared to MCMV-BAC. **(E)** Enumeration of MCMV-specific m78-Tetramer-positive CD4 T cells upon MCMV-B5 compared to MCMV-BAC from day 15 MCMV-infected mice. Plots represents control CLIP tetramer or m-78-tetramer staining after MCMV-B5. Graph shows number of MCMV-specific m78 Tet+ T cells (±SEM). **(F)** Enumeration of MCMV-specific m45-Tetramer-positive CD8 T cells. Mice were infected with MCMV-B5 and splenocytes were recovered 11 and 60 days later for CD8 T cell quantification by staining with m45 tetramers. Plots and graph show m45-Tet^{+} $CD8^{+}$ T cells from uninfected and MCMV-B5 infected mice gating on vector specific T cells (+/- SEM). Data shown are representative of two experiments with 3 or 5 mice each. Groups were analyzed using One-Way ANOVA followed by Tukey's post-test with *, $P < 0.05$; **, $P < 0.01$.

S2 Fig. Persistent MCMV-B5 infection induces vector-specific T cell response and may promote continued B5 TCR Tg T cell proliferation.

(A) Experimental schematic shows mice were infected with MCMV-B5, and splenocytes were recovered 11 or 60 days later to quantify MCMV m-78-specific $Tetramer^{+}$ CD4 T cells. (**B**) Plots and graph show uninfected and m78 tetramer-specific T cell numbers. Virus-specific $CD4^{+}$ T cells were identified using m78/I-A^{d} tetramers, with peptide (SQQKMTSLPMSVFYS) and CLIP/I-A^{d} as tetramer control (PVSKMRMATPLLMQA). (**C**) Plots and graphs show the number of MCMV-specific-Teff ($CD44^{+}CD127^{-}$) and Tmem ($CD44^{hi}CD127^{hi}$) in the spleen gated on MCMV m78-tetramer $CD4^{+}$ T cell at day 11 and 60 p.i.. Data shown are representative of two experiments with 5 mice each and error bars representing SEM. **(D)** Experimental Schematic shows MCMV-BAC or MCMV-B5 infection of different groups of age-matched Thy1.1 mice at indicated times (0, 30

bioRxiv preprint doi: https://doi.org/10.1101/2023.05.02.539025; this version posted May 16, 2023. The copyright holder for this preprint (which was not certified by peer review) is the author/funder, who has granted bioRxiv a license to display the preprint in perpetuity. It is made available under aCC-BY 4.0 International license.

and 50 days) followed by adoptive transfer of CTV$^+$ B5 TCR Tg CD4 T cells to all groups 5 days before phenotyping, and 55 days after first infection. **(E)** Plots and graphs showing gating and frequency of CTV-B5 T cells out of CD4 T cells, or number of proliferated B5 epitope-specific CD4 T cells in response to MCMV-BAC or MCMV-B5 infection at different time points. Data are representative of three independent experiments with 5 animals per group. Mean shown with error bars representing SEM. Groups were analyzed using One-Way ANOVA followed by Tukey's post-test with *, $P < 0.05$; **, $P < 0.01$; ***, $P < 0.001$.

S3 Fig. Decay of *P. chabaudi* protection by days 200

(A) Experimental schematic shows age-matched mice were infected with *P. chabaudi* AS for assessment of protection to heterologous challenge with *P. chabaudi* AJ 60 or 200 days later. Graphs show **(B)** the percentage of parasitemia (iRBC/RBC) after heterologous challenge, and **(C)** *P. chabaudi* AJ-specific IgG antibody serum level at day 7 post-challenge. Data are representative of two independent experiments with five mice per group showing mean, with error bars representing SEM. Groups were analyzed using One-Way ANOVA followed by Tukey's post-test with *, $P < 0.05$; **, $P < 0.01$; ***, $P < 0.001$.

S4 Fig: Evaluation of neutralizing effect of anti-IFN-γ antibody

(A) Experimental schematic shows mice were infected or not infected with MCMV-BAC to assess the neutralizing effect of anti-IFN-γ naïve mice or in MCMV-induced protection to *P. chabaudi*. Blocking of IFN-γ *in vivo* with anti-IFN-γ antibody or isotype control antibody was performed at days 34, 36 and 38 prior to the challenge or infection with *P. chabaudi* at day 40 in both MCMV infected and naïve mice. Graphs show percentage of parasitemia in **(B)** no MCMV mice and **(C)**

bioRxiv preprint doi: https://doi.org/10.1101/2023.05.02.539025; this version posted May 16, 2023. The copyright holder for this preprint (which was not certified by peer review) is the author/funder, who has granted bioRxiv a license to display the preprint in perpetuity. It is made available under aCC-BY 4.0 International license.

MCMV vaccinated mice**.** Relative CXCL-10 mRNA level normalized with β-actin from Raw 264.7 cells cultured for 24h with sera from: (**D**) 2 days after anti-IFN-γ administration, (**E**) day 7 post *P. chabaudi* challenge, (**F**) 2 days after anti-IFN-γ administration in presence of rIFN-γ (25ng/ml), and (**G**) day 7 post *P. chabaudi* challenge administration in presence of rIFN-γ (25ng/ml). Data shown represent 5 animals per group and mean shown with error bars representing SEM. Groups were analyzed using Student *t* test (C, D and F) or One-Way ANOVA (B, E and G) with *, $P < 0.05$; **, $P < 0.01$; ***, $P < 0.001$.

S5 Fig. Combined IL-12 and IL-18 neutralization did not affect protection induced by MCMV.

(A) Experimental schematic shows mice infected with MCMV-BAC and the combination of anti-IL-12p40 and anti-IL-18 antibody or isotypes were given post MCMV infection. Graphs show percentage of parasitemia **(B)**, weight loss **(C)**, and hypothermia **(D)** upon combined IL-12 and IL-18 neutralization. Data shown represent 5 animals per group and mean shown with error bars representing SEM and analyzed using multiple comparisons Student *t* test; n.s., not significant.

S6 Fig. IFN-γ induced by MCMV vector does not change most antigen-presenting cells types and their activation significantly.

(A) Experimental schematic shows mice infected with MCMV-B5 and received anti-IFN-γ antibody or isotype at indicated time points. Splenic antigen-presenting cells types (monocytes, macrophages and inflammatory monocytes) and NK cells were phenotyped at day 50 p.i.. **(B)** Plots and graphs show gating and quantification of $CD3^{-}CD11b^{-}Ly6C^{+}$, $CD3^{-}$ $CD11b^{+}Ly6C^{+}$, $CD3^{-}$ $CD11b^{+}Ly6C^{-}$ cells. (C) Histograms and graphs show MFI of CD3- CD11b+Ly6C-

bioRxiv preprint doi: https://doi.org/10.1101/2023.05.02.539025; this version posted May 16, 2023. The copyright holder for this preprint (which was not certified by peer review) is the author/funder, who has granted bioRxiv a license to display the preprint in perpetuity. It is made available under aCC-BY 4.0 International license.

expressing activation marker MHCII. (D) Histograms and graphs show MFI of CD3-CD11b+Ly6C- expressing activation marker CD86. (**E**) Plots and graphs show gating and quantification of CD3$^-$ DX5$^+$MHCII$^-$ NK cells.

(**F**) Experimental schematic shows Thy1.1 mice vaccinated with live malaria vaccine. Then vaccinated mice received MCMV-B5 boosters at day 140 and then received anti-IFN-γ antibody or isotype at indicated days (194, 196, 198) before spleen cell recovery for cells phenotyping at day 200 p.v. **(G)** Plots and graphs show gating and quantification of CD3$^-$ CD11b$^-$Ly6C$^+$, CD3$^-$ CD11b$^+$Ly6C$^+$, CD3$^-$ CD11b$^+$Ly6C$^-$ cells. **(H)** Histograms and graphs show MFI of macrophages (CD3$^-$CD11b$^+$Ly6C$^-$) expressing activation marker MHCII. **(I)** Histograms and graphs show MFI of inflammatory monocytes (CD3$^-$ CD11b$^+$Ly6C$^+$) expressing activation marker MHCII. (**J**) Plots and graphs show gating and quantification of CD3$^-$DX5$^+$MHCII$^-$ NK cells. Data shown are representative 5 mice per group. Plots concatenated per group and mean shown with error bars representing SEM. The Student *t* test or One-Way ANOVA were used.

S7 Fig. An example of a gating strategy used to identify polyclonal CD4$^+$ T cells subsets and B5-specific CD4$^+$ T cells subsets

(Top row) Plots show gating strategy for polyclonal CD4$^+$ T cell subsets Teff (CD4$^+$CD127$^-$CD44^{hi}), Tmem (CD4$^+$CD127^{hi}CD44^{hi}); and memory T cell subsets gated on CD44^{hi}CD127^{hi} as Tcm (CD62L$^+$CD27$^+$), Tem^{Early} (TemE, CD62L^{lo}CD27$^+$) or Tem^{Late} (TemL, CD62L^{lo}CD27$^-$).

(Bottom row) Plots show gating strategy for B5-specific T cells: Teff (CD4$^+$Thy1.2$^+$CTV$^-$, CD127$^-$CD44^{hi}), Tmem (CD4$^+$Thy1.2$^+$CTV$^-$, CD127^{hi}CD44^{hi}) and B5 memory T cell subsets gated on CD44^{hi}CD127^{hi}, Tcm (CD62L$^+$CD27$^+$), Tem^{Early} (TemE, CD62L^{lo}CD27$^+$) or Tem^{Late} (TemL, CD62L^{lo}CD27$^-$).
